# Increased risk of early-onset childhood systemic lupus erythematosus for children born to affected parents: A nationwide child-parent cohort study

**DOI:** 10.3389/fimmu.2022.966809

**Published:** 2022-09-05

**Authors:** Chun-Hsin Wu, Chih-An Chen, Sheng-Hsiang Lin, Chia-Tse Weng, Pao-Lin Kuo, Chi-Chang Shieh

**Affiliations:** ^1^ Institute of Clinical Medicine, College of Medicine, National Cheng Kung University, Tainan, Taiwan; ^2^ Division of Allergy, Immunology and Rheumatology, Department of Internal Medicine, National Cheng Kung University Hospital, College of Medicine, National Cheng Kung University, Tainan, Taiwan; ^3^ Department of Pediatrics, National Cheng Kung University Hospital, College of Medicine, National Cheng Kung University, Tainan, Taiwan; ^4^ Department of Public Health, College of Medicine, National Cheng-Kung University, Tainan, Taiwan; ^5^ Biostatistics Consulting Center, National Cheng Kung University Hospital, College of Medicine, National Cheng Kung University, Tainan, Taiwan; ^6^ Department of Obstetrics and Gynecology, National Cheng Kung University Hospital, College of Medicine, National Cheng Kung University, Tainan, Taiwan

**Keywords:** childhood-onset systemic lupus erythematosus, maternal systemic lupus erythematosus, paternal autoimmune diseases, child-parent linkage, epidemiology - analytic (risk factors)

## Abstract

**Objective:**

Children of women with systemic lupus erythematosus (SLE) are at risk for childhood-onset SLE (cSLE). This study evaluated the incidence of early-onset cSLE and associated risk factors, including concomitant maternal and paternal autoimmune diseases, for these children.

**Methods:**

A population-based cohort study was conducted using national databases including the linked information of children and parents. Children of women with SLE and those of women without SLE were identified between 2004 and 2015. The cumulative cSLE incidence was estimated using the Kaplan-Meier method. The marginal Cox model was used to calculate the hazard ratio (HR) for cSLE events.

**Results:**

A total of 4,419 singletons of women with SLE and 1,996,759 singletons of women without SLE were identified. There were 9 (0.20%) and 503 (0.03%) incident cases of early-onset cSLE for offspring of women with and without SLE, respectively (incidence rate ratio, 8.34; 95% confidence interval [CI], 3.79–15.95]. The adjusted HR of incident cSLE in children of women with SLE was 4.65 (95% CI 2.11–10.24). Other risks for cSLE included pregnancy-induced hypertension/preeclampsia/eclampsia, paternal SLE, paternal Sjögren’s syndrome (SS), and maternal SS.

**Conclusions:**

This national child-parent cohort study demonstrated that children of women with SLE are at significantly higher risk for cSLE during early childhood. Moreover, paternal SLE and parental SS increase the risk of cSLE for offspring.

## Introduction

Systemic lupus erythematosus (SLE) is a potentially fatal, chronic autoimmune disorder that involves many organs (1). A combination of genetic, environmental, and hormonal factors contributes to the loss of immune tolerance, and the secretion of antibodies against self-antigens is a hallmark of SLE pathogenesis (2). The estimated prevalence of SLE in Taiwan is 4.8–8.1 per 10,000 individuals (3) and 2.2–10.3 per 10,000 individuals in North America (4). SLE predominantly affects women during childbearing years. It is well established that autoimmune diseases exhibit familial aggregation, and early family studies have found higher SLE incidence rates for monozygotic twins (24%–56%) than for dizygotic twins (2%–5%) (5). Indeed, it has been shown that individuals with affected first-degree relatives have a 17-fold increased risk of SLE (6).

Several studies have found that women with SLE are at an increased risk for adverse obstetric outcomes, such as premature birth or newborns who are small for gestational age (7), and for birthing offspring at risk for subsequent immune or nonimmune-mediated morbidity during childhood (5, 8–13). Neonatal lupus erythematosus (NLE) is an acquired autoimmune disease that occurs in newborns of women with anti-Ro and/or anti-La autoantibodies (14). The clinical course of NLE is predominantly mild, transient, and self-limited. When lupus occurs in individuals who are younger than 18 years old, it is referred to as childhood-onset SLE (cSLE), which is a rare disease with a reported prevalence of approximately 0.3–2.4 per 10,000 children (15). The prevalence of lupus nephritis and end-stage renal disease in cSLE is higher than that in adult-onset SLE (aSLE) (16). In particular, the mortality rate is higher in early-onset cSLE compared with later-onset cSLE (17).

Few studies have evaluated the risk of autoimmune diseases during the growth period of children of women with SLE. A recent large cohort study performed in Québec, Canada, revealed that the offspring of women with SLE were at greater risk for nonrheumatic autoimmune disease than for rheumatic autoimmune diseases (18), which is contrary to common knowledge. Epidemiological data regarding early-onset cSLE (onset between ages older than 2 years and younger than 13 years) is even more limited. Previous studies have been limited by small sample sizes, cross-sectional design, and single-center analyses. Furthermore, no studies have investigated the influence of concomitant paternal autoimmune diseases on children. Therefore, we conducted this large-scale, population-based study to explore the risk of cSLE among children of women with SLE.

## Materials and methods

### Study design, data source, and ethical approval

A longitudinal, retrospective, nationwide cohort study was conducted. Population-based data were retrieved from the Birth Reporting Database (BRD), Taiwan Maternal and Child Health Database (TMCHD), and the National Health Insurance Research Database (NHIRD) provided by the Health and Welfare Data Science Center of the Ministry of Health and Welfare. All live births and stillbirths with birth weights >500 g or gestational ages >20 weeks are registered in the BRD. The TMCHD provides the parent-child kinship for each infant. The NHIRD contains reimbursement claims data from the National Health Insurance, a compulsory insurance program launched in 1995 that provides 99% coverage of 23 million individuals in Taiwan. This healthcare system incorporates demographic data, diagnostic codes, ambulatory and inpatient medical expenditures, and surgery codes. All beneficiaries have encryption numbers that are used to perform linkage within these national databases. This methodology for interdatabase linkage has been well-validated by several research articles (19–21). The Institutional Review Board of the National Cheng Kung University Hospital approved the study protocol (A-ER-108-245). This study complied with the Declaration of Helsinki, and the requirement for written informed consent for data analysis was waived because of the retrospective nature of the study. All data were encrypted before being released for research purposes.

### Study population

All live singletons listed in the BRD with a birthdate between January 1, 2004 and December 31, 2015 were identified and followed up until December 31, 2017. Parents of each newborn were identified using the TMCHD and linked within the NHIRD between January 1, 2000 and December 31, 2017. The index date was defined as the date of childbirth. Children exposed to maternal SLE were identified by interlinking databases. True *in utero* exposure to SLE was considered when pregnant women received an SLE diagnosis code within 4 years before the index date, and their children were categorized as offspring of women with SLE. If a pregnant woman was diagnosed with SLE prior to the 4 years before the index date but did not receive the corresponding code within the 4 years before the index date, the status for SLE is considered either inactive or misclassified. We included women who had concomitant autoimmune diseases, which were designated as comorbidities, before the index date in this study.

### Case definition of SLE

SLE was identified by the International Classification of Diseases, Ninth Revision, Clinical Modification codes (710.0) for dates before December 31, 2015, and by the International Classification of Diseases, Tenth Revision, Clinical Modification codes (M32.1-M32.9) for dates after December 31, 2015. We validated a true diagnosis of SLE only if there were catastrophic illness certificates (CICs) for SLE. The CICs were issued by the Administration of National Health Insurance only when two independent rheumatologists ascertained the corresponding disease classification criteria for SLE (22) after reviewing the medical records, laboratory data, and imaging results.

### Outcome of interest

The primary outcome of the current study was incident early-onset cSLE. In Taiwan, the average age for puberty is 12.32 ± 1.22 years for boys and 11.35 ± 1.06 years for girls (23). Therefore, we defined the age of onset between >2 years and <13 years as pre-pubertal, early-onset cSLE in the current study. In contrast, NLE was considered when the age of onset was younger than 6 months; these individuals were not counted as cSLE cases. All children were followed up from the index date until the occurrence of the primary outcome or the end date of the study (December 31, 2017), whichever came first.

### Covariates and potential confounders

Demographic information of neonates, including sex, fetal gestational age, and birth weight for gestational age, were retrieved from the BRD. The age of each parent was determined using their date of birth and the child’s date of birth. We included obstetric complications as potential confounders, including pregnancy-induced hypertension (PIH), preeclampsia, eclampsia, and gestational diabetes. Rheumatoid arthritis (RA), Sjögren’s syndrome (SS), Graves’ disease, Hashimoto thyroiditis, and type 1 diabetes mellitus were selected as concomitant paternal or maternal autoimmune diseases influencing cSLE. These comorbid immune diseases were determined during the 2-year period before the index date. Participants were considered to have a specific comorbidity only if they had the corresponding diagnostic code at least three times in their outpatient claims or at least once in their inpatient claims (for a full list of diagnostic codes, see **Supplementary Table S1**). Income and housing urbanization levels were used to identify the socioeconomic status of individuals. Family income level was categorized into quartiles by summing the monthly income of the parents. Urbanization levels were defined according to a previous study (24).

### Statistical analysis

Continuous variables with normal distribution were expressed as the mean and standard deviation and categorical variables as numbers and percentages. Student’s t-test or Pearson’s chi-squared test was used for between-group comparisons, as appropriate. Incidence rates were estimated by dividing the cSLE events observed by the person-years contributed by individuals during the observation period, and the incidence rate ratios were calculated by Poisson regression. The cumulative incidences of cSLE for children were illustrated using the Kaplan-Meier method, and the log-rank test was used to compare between-group differences. To assess the relative risk of cSLE among children of women with SLE, a multivariable Cox proportional hazard regression model adjusted for potential confounders including maternal age, paternal age, obstetric characteristics (gestational age category, birthweight category), obstetric complications (PIH/preeclampsia/eclampsia, and gestational diabetes), mode of delivery, offspring sex, concomitant paternal autoimmune diseases (paternal SLE, maternal/paternal SS, maternal RA, maternal Graves’ disease), and socioeconomic parameters (income and urbanization level) was used. Additional analyses were performed to assess the relative risk of cSLE associated with covariates independently of maternal SLE. Multivariable Cox models were adjusted for maternal SLE and the aforementioned covariates, except for the variable itself. Because some women may give birth to more than one child, the marginal approach with robust sandwich covariance matrix estimation was applied to derive adjusted hazard ratios (HR) among these dependent samples (25). In the univariable and multivariable Cox regression models, individuals were excluded from the analyses if they had any missing variables. All statistical analyses were performed using SAS version 9.4 (SAS Institute, Cary, NC, USA). A two-sided *p* < 0.05 was considered statistically significant.

### Sensitivity analysis

During the primary analysis, the index date was defined as the date of childbirth, and the outcome was not measured until the participants were 2 years of age. This may have contributed to immortal time bias. To appraise the robustness of our results, we conducted a sensitivity analysis by changing the index date to reflect a child 2 years of age.

## Results

### Clinical characteristics of pregnant women with SLE

**Figure 1:**
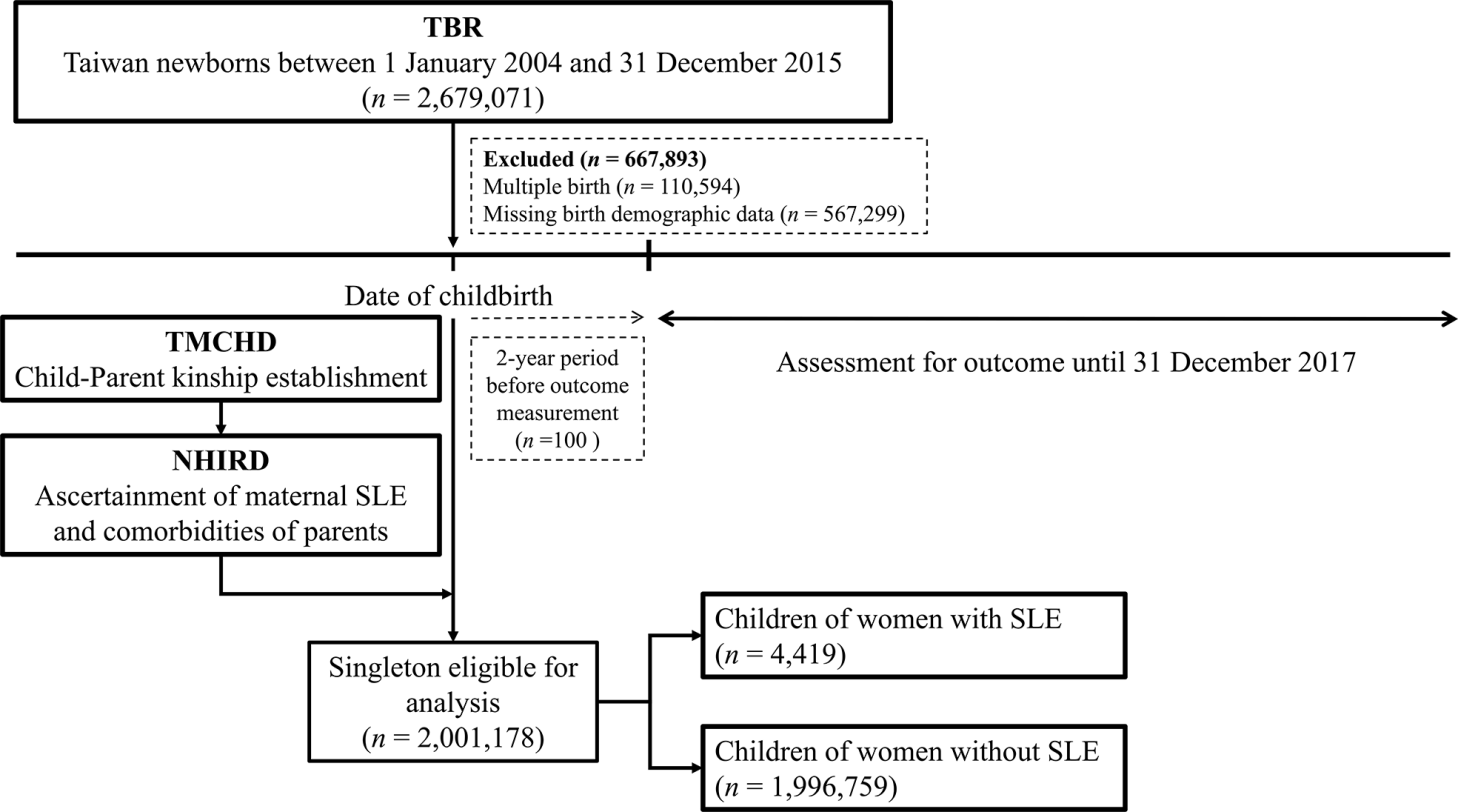
Flow chart of the study design.

A total of 4,419 singletons of women with SLE and 1,996,759 singletons of women without SLE were identified between 2004 and 2015 (**Figure 1**). Baseline information of the family triads (mother, father, and child) is summarized in **Table 1**. The mean ages of the women with SLE and their spouses were similar to the ages of those without SLE. Women with SLE experienced substantially more preterm births (<37 weeks of gestation) and more often birthed newborns who were small for their gestational age. Compared to women without SLE, women with SLE experienced more obstetric complications, such as PIH/preeclampsia/eclampsia, and gestational diabetes. Additionally, more pregnant women with SLE required cesarean delivery compared to women without SLE. The sex ratio of the offspring was balanced among both groups. Women with SLE had more concomitant autoimmune diseases, such as SS, RA, autoimmune thyroiditis, and type 1 diabetes, than women without SLE.

**Table 1:** Baseline characteristics of mothers with and without SLE and their singletons born between 2004 and 2015 identified in the Taiwan.

	Mother without SLE		Mother with SLE	
**Variable**	**(n = 1,996,759)**		**(n = 4,419)**	
**Maternal characteristics**				
**Age,** mean ± SD, years	30.18 ± 4.82		31.37 ± 4.43	
**Maternal age group (years),** n (%)				
<20	40115	(2.01)	28	(0.63)
20-24	246639	(12.38)	321	(7.27)
25-29	661799	(33.22)	1307	(29.58)
30-34	733855	(36.80)	1854	(41.96)
35-39	270876	(13.60)	797	(18.04)
≥40	38907	(1.95)	111	(2.51)
Missing data	4568		1	
**Paternal characteristics**				
**Age,** mean ± SD, years	33.18 ± 5.38		33.66 ± 4.97	
**Paternal age group (years),** n (%)				
<24	96287	(5.11)	139	(3.29)
25-29	421792	(22.37)	801	(18.94)
30-34	734081	(38.93)	1734	(41.00)
35-39	447654	(23.74)	1141	(26.98)
40-44	140655	(7.46)	340	(8.04)
45-49	34014	(1.80)	51	(1.21)
≥50	11103	(0.59)	23	(0.54)
Missing data	111173		190	
**Obstetric characteristics**				
**Gestational age category (weeks), n (%)**				
<31	11058	(0.55)	99	(2.24)
31-33	11578	(0.58)	90	(2.04)
34-36	117410	(5.88)	552	(12.50)
≥37	1856713	(93.00)	3678	(83.23)
**Mean gestational age,** mean ± SD, weeks	38.39 ± 1.53		37.75 ± 2.13	
**Birthweight for gestational age, n (%)**				
SGA	192417	(9.64)	796	(18.01)
AGA	1596063	(79.93)	3329	(75.33)
LGA	208272	(10.43)	294	(6.65)
Missing data	7		0	
PIH/preeclampsia/eclampsia	49150	(2.46)	259	(5.86)
Gestational diabetes	260958	(13.07)	689	(15.59)
**Mode of delivery,** n (%)				
Normal spontaneous delivery	1315667	(65.89)	2500	(56.6)
Cesarean delivery	681092	(34.11)	1919	(43.4)
**Offspring sex,** n (%)				
Male	1040675	(52.12)	2278	(51.55)
Female	956077	(47.88)	2141	(48.45)
Paternal SLE	633	(0.03)	8	(0.18)
Maternal Sjögren’s syndrome	7103	(0.36)	925	(20.93)
Paternal Sjögren’s syndrome	2961	(0.15)	22	(0.50)
Maternal rheumatoid arthritis	3746	(0.19)	355	(8.03)
Paternal rheumatoid arthritis	2906	(0.15)	9	(0.20)
Maternal Graves’ disease	15521	(0.78)	77	(1.74)
Paternal Graves’ disease	4347	(0.22)	16	(0.36)
Maternal Hashimoto thyroiditis	4386	(0.22)	80	(1.81)
Maternal type 1 diabetes	1302	(0.07)	8	(0.18)
**Family income level,** n (%)				
Dependent or <Q1	467538	(23.41)	743	(16.81)
Q1 to <Q2	531431	(26.61)	1044	(23.63)
Q2 to <Q3	502477	(25.16)	1214	(27.47)
≥Q3	495313	(24.81)	1418	(32.10)
**Urbanization level,** n (%)				
Level 1 or 2	154733	(7.75)	281	(6.36)
Level 3 or 4	317872	(15.90)	713	(16.13)
Level 5	466566	(23.40)	906	(20.50)
Level 6	623058	(31.20)	1561	(35.32)
Level 7	434530	(21.76)	958	(21.68)

SLE, systemic lupus erythematosus; SGA, small for gestational age; AGA, appropriate for gestational age; LGA, large for gestational age; PIH, pregnancy-induced hypertension; SD, standard deviation; Q, quartile. Family income level, Q1 denotes the lowest monthly income amount and Q3 denotes the highest monthly income amount. Urbanization level, level 1 denotes most urbanized and level 7 denotes least urbanized.Maternal and Child Health Database.

### Incidence of cSLE among the study population

**Figure 2:**
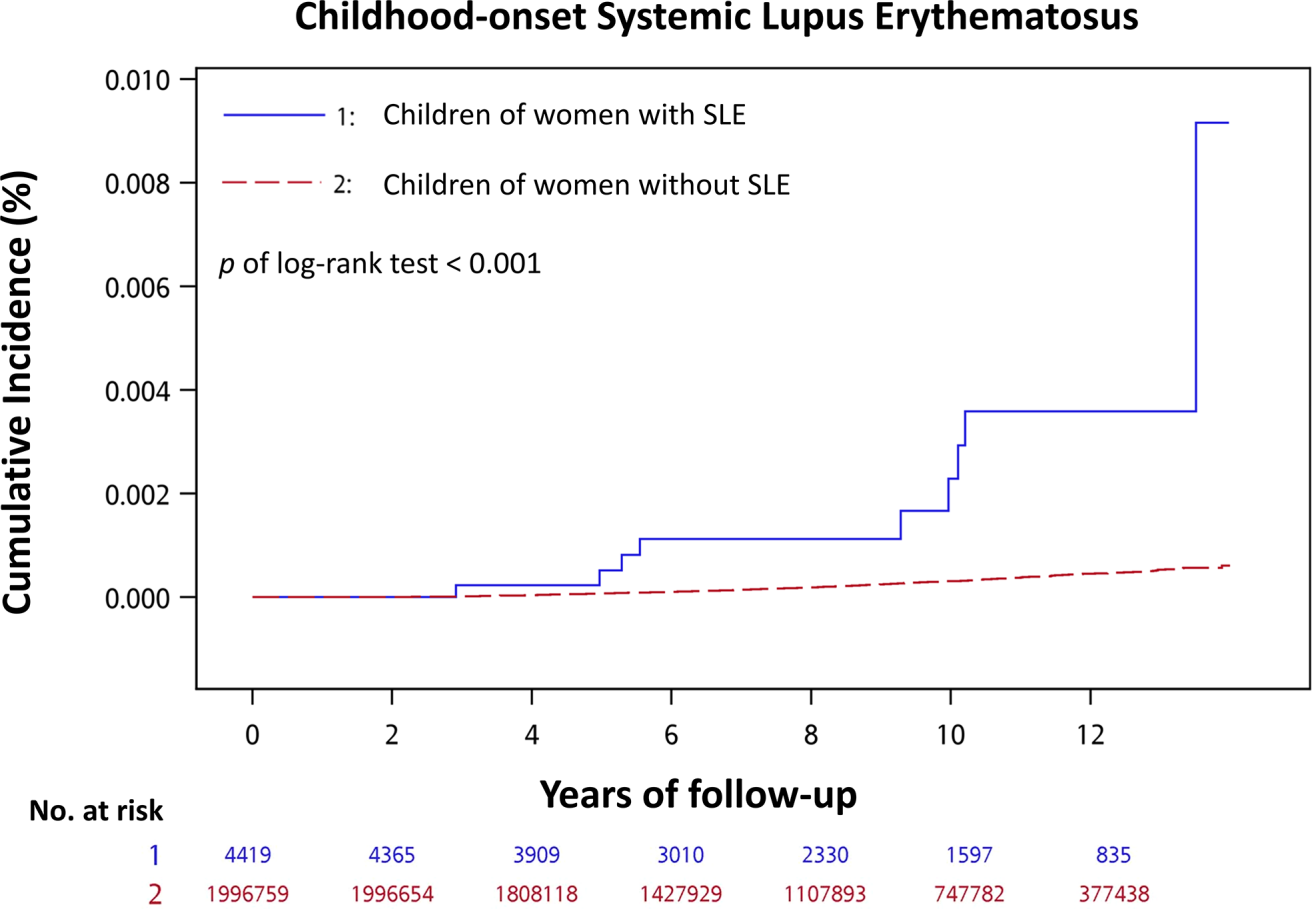
Cumulative incidence of childhood-onset systemic lupus erythematosus for children of women with SLE compared with children of women without SLE.

Among children of women with SLE and those of women without SLE, 9 (0.20%) and 503 (0.03%) developed cSLE during an approximately 8-year median follow-up period, respectively (**Table 2**). The mean age when cSLE was diagnosed was 7.98 ± 3.42 years for children of women with SLE, and it was 7.36 ± 2.89 years for children of women without SLE in the control group (**Table 2**). The incidence rate (per 1000 person-years) of cSLE was 0.322 for children of women with SLE, and it was 0.039 for children of women without SLE The cSLE incidence rate ratio for the children of women with SLE compared with those of women without SLE was 8.34 (95% CI, 3.79–15.95). The cumulative incidence of cSLE was significantly higher for children of women with SLE than for children of women without SLE (log-rank test, *p* < 0.001) (**Figure 2**).

**Table 2:** Outcomes of offspring of women with SLE compared with those of women without SLE.

	Children of mothers without SLE		Children of mothers with SLE		*p* value
**Median follow-up duration, (**25%-75% IQR), **years**	8.59 (5.65–11.32)		8.26 (5.36–11.29)		<0.001
**Ealy-onset cSLE**, n (%)	503 (0.03)		9 (0.20)		<0.001
**Disease-onset age** (years), mean ± SD	7.36 ± 2.89		7.98 ± 3.42		0.525
**Incidence rate of early-onset cSLE (1000 person-years)**	0.039		0.322		
**Incidence rate ratio of early-onset cSLE (95% CI)**	1.0 (reference)		8.34 (3.79–15.95)		<0.001

IQR, interquartile range; SLE, systemic lupus erythematosus; cSLE, childhood-onset SLE; SD, standard deviation; CI, confidence interval.

### Relative risk of childhood-onset SLE

**Table 3** illustrates the results of the multivariable Cox regression model adjusted for potential confounders, including maternal age, paternal age, obstetric characteristics, obstetric complications, mode of delivery, offspring sex, concomitant paternal autoimmune diseases, and socioeconomic parameters. In the partially adjusted model, obstetric complications (PIH/preeclampsia/eclampsia, and gestational diabetes) were designated as mediators and were not adjusted. Children of women with SLE had a significantly higher risk of cSLE (adjusted 4.81; 95% CI, 2.17–10.65 and fully adjusted HR, 4.65; 95% CI, 2.11–10.24, respectively). The relative risk of those covariates for developing cSLE independent of maternal SLE was estimated by additional multivariable Cox regression models, and the results are presented in **Supplementary Table S2**. Female sex (adjusted HR, 2.46; 95% CI, 2.03–2.98), having a father with SLE (adjusted HR, 12.95; 95% CI, 2.66–63.16), maternal SS (adjusted HR, 3.12; 95% CI, 1.39–7.00), and paternal SS (adjusted HR, 4.68; 95% CI, 1.59–13.93) were factors associated with an increased risk of cSLE. In contrast, maternal RA did not increase the risk of cSLE. The fully adjusted HR of the above-mentioned covariates was presented in **Supplementary Table S2** (right panel), and the results remained at a similar magnitude. Furthermore, preterm birth, small or large for gestational age, and gestational diabetes mellitus were not risk factors for cSLE.

**Table 3:** Crude and adjusted hazard ratios of maternal SLE leading to early-onset childhood SLE.

	Maternal SLE	*p* value
**Crude HR (95% CI)**	8.34 (4.32–16.12)	<0.001
Adjusted HR^a^ (95% CI)	4.81 (2.17–10.65)	<0.001
Fully adjusted HR^b^ (95% CI)	4.65 (2.11–10.24)	<0.001

SLE, systemic lupus erythematosus; CI, confidence interval; HR, hazard ratio.

a Adjusted for maternal and paternal age, obstetric characteristics (gestational age category, birthweight category), mode of delivery, offspring sex, concomitant paternal autoimmune diseases, and socioeconomic parameters (income and urbanization level) listed in **Table 1** but excluding the variable of obstetric complications (PIH/preeclampsia/eclampsia, and gestational diabetes).

b Adjusted for maternal and paternal age, obstetric characteristics (gestational age category, birthweight category), obstetric complications (PIH/preeclampsia/eclampsia, and gestational diabetes), mode of delivery, offspring sex, concomitant paternal autoimmune diseases, and socioeconomic parameters (income and urbanization level) listed in **Table 1**.

### Sensitivity analysis

After changing the index date to 2 years after childbirth, we found that the incidence rate ratio and relative risk of cSLE remained higher among children of women with SLE (adjusted HR, 5.43; 95% CI, 2.40–12.26) (**Supplementary Table S3**).

## Discussion

To the best of our knowledge, the present study is the first nationwide cohort study to assess the risk of early-onset cSLE among children of women with SLE. Our major finding shows that children of women with SLE exhibit a significantly increased risk of cSLE compared to children of women without SLE.

Parents and children share a similar genetic predisposition and environmental exposure to SLE; therefore, familial aggregation is highly possible. A single-hospital cohort study conducted in India estimated the prevalence of autoimmune rheumatic diseases at 24.8% for families of individuals with SLE (13). Among the affected family members, SLE was most commonly observed in both first-degree and second-degree relatives. Furthermore, a nationwide study performed in Taiwan revealed that the relative risk of SLE was highest for twins of individuals with SLE, followed by siblings, parents, and offspring of individuals with SLE (6). Interestingly, during a more recent family study performed in Brazil that evaluated both cSLE and aSLE, the authors found a significantly higher SLE recurrence rate for first-degree and second-degree relatives of individuals with cSLE than for those of individuals with aSLE (26). However, none of these studies considered the timing of the SLE diagnosis for both individuals with SLE and their families; hence, the risk of cSLE during childhood cannot be evaluated. In contrast, using integrated national databases indicating the precise chronological order of the SLE diagnosis for participants, we observed an eightfold increased incidence of cSLE for the offspring of women with SLE.

A complex mechanism involving genetic susceptibility, epigenetic modifications, and environmental triggers contributes to the pathogenesis of SLE. Subsequently, impaired immune homeostasis including dysregulated apoptosis, decreased clearance of nuclear debris, aberrations of type 1 interferon, autoreactive B cell and T cell activation, and production of autoantibodies with immune complex formation can lead to overt systemic inflammation (2). Both cSLE and aSLE are considered polygenic disorders, and fundamental genetic factors cause SLE to have a strong familial aggregation, with an estimated heritability of approximately 43.9%–66% among different cohorts (6, 27). However, the concordance rates of SLE for monozygotic twins are <60%, and they are even lower for dizygotic twins (28), implying that epigenetic and environmental factors contribute to the development of SLE. DNA hypomethylation, histone modifications, and noncoding RNA regulation are three major epigenetic mechanisms that may be inherited from the parents or occur randomly because of environmental triggers (29). Ultraviolet B light exposure, Epstein-Barr virus infection, and estrogen exposure are the most common environmental triggers; to a lesser extent, dietary habits and smoking are possible environmental factors that promote SLE initiation.

The Offspring of SLE Mothers Registry, a large Canadian cohort of women with SLE, and a matched group were involved in a study performed between 1989 and 2009 that provided epidemiologic information about offspring of women with SLE (12, 17). Contradicting our results, the Offspring of SLE Mothers Registry study did not observe an increased risk of rheumatic autoimmune diseases, and there were no cSLE events among 719 children of women with SLE. This discrepancy may be attributed to several reasons. Firstly, the overall SLE incidence rate is higher for Asian ethnicity than for White ethnicity (4). Secondly, epidemiological studies have shown that cSLE is rarer than aSLE (30, 31). We identified 4,419 children of women with SLE during our study. This is the largest parent-child cohort in the current literature. Therefore, with the appropriate follow-up duration, we can assess the risk of cSLE.

Childhood-onset SLE can be further classified into early-onset (<13 years) and later-onset (≥13 years) cSLE. Studies have shown that patients with early-onset cSLE have a higher intensive care unit admission rate and mortality rate compared to patients with later onset cSLE (17, 32). In our 13-year cohort study, the incidence of early-onset cSLE was significantly higher among children of women with SLE. In addition to genetic or immunological mechanisms contributing to a higher risk of early-onset cSLE, parents with SLE are more aware of autoimmune diseases and more likely to have their child diagnosed earlier. Nevertheless, we found a similar mean age of disease onset for children of mothers with and without SLE. These findings remind physicians to provide timely and optimal care for these vulnerable patients.

Fathers genetically contribute to the offspring in a nearly equal manner to mothers; however, studies addressing the impact of paternal autoimmune diseases on children are scarce because the prevalence of male autoimmune diseases during childbearing age is low. According to a population-based parent-child study performed in Denmark, among offspring with SLE, the SLE histories of their mothers and fathers contributed nearly the same risk (33). However, we found that compared with a positive maternal history, a positive paternal history of SLE was associated with a greater risk of cSLE among offspring. A possible reason for these conflicting results was that the Danish cohort included a substantial number of aSLE cases among offspring during a 33-year follow-up period. In contrast, our study focused on the risk of incident cSLE among children with affected parents. Further large-scale prospective research is necessary to compare the different magnitudes of risk factors of fathers and mothers.

Few studies have examined the outcomes of children with parents with SS beyond the perinatal period, and most of the previous studies have focused on the risks of NLE and congenital heart block for neonates of women with SS (34). Transplacental passage of anti-Ro or anti-La antibodies causes tissue inflammation in fetuses and newborns (14). Approximately 60%–70% of individuals with SS and 30%–40% of individuals with SLE possess these autoantibodies. However, long-term follow-up of children with parents with SS is lacking. Kuo *et al.* reported various increased autoimmune disease phenotypes of relatives of individuals with SS (35); however, the timing of the SS diagnosis for participants and the timing of autoimmune disease diagnoses for their relatives were not considered chronologically. In this study, we designated paternal SS and maternal SS as covariates and found that both increased the risk of cSLE development among offspring. The results of our study clearly support the concept that SLE and SS share similar immunopathogenesis. Recent genome-wide association studies have identified several susceptible risk loci for both SLE and SS (36). Additionally, overactive plasmacytoid dendritic cells and enhanced type I interferon signatures have been observed in SLE and SS (37).

PIH/preeclampsia/eclampsia comprise a spectrum of hypertensive disorders of pregnancy; it has been established that excessive maternal systemic inflammatory response involves its pathogenesis (38, 39). Several observational studies have revealed that preeclampsia is associated with an increased risk of allergic respiratory diseases for offspring (40–42). In the fully adjusted Cox regression model designating PIH/preeclampsia/eclampsia as a confounder for cSLE, we further found that PIH/preeclampsia/eclampsia was associated with a higher risk of developing cSLE during childhood after adjustment of confounding covariates. Our findings elicit a hypothesis that maternal immune activation and placental environmental triggers contribute to susceptibility for cSLE.

Before pregnancy, any teratogenic drug should be stopped to optimize fetal health. Several immunosuppressive agents, including glucocorticoid, hydroxychloroquine, and azathioprine, are permitted to control SLE during pregnancy (43). The common use of these drugs in pregnant SLE women leads to the legitimate concern that prenatal medication exposure may affect the disease development in children. While hydroxychloroquine exposure improves neonatal outcomes (44, 45), most of the literature regarding *in utero* medication exposure among offspring of pregnant SLE women focused on postnatal immunodeficiency, neurodevelopmental deficits, or congenital anomalies (46–48). However, long-term follow-up of children exposed *in utero* to immunosuppressive drugs was limited. In the present study, we could not assess the potential impact of antenatal immunosuppressant exposure among offspring of women with SLE due to the lack of access to a medication dataset and low incidence of cSLE. Further large-scale prospective studies with longer follow-up duration are certainly needed to unravel the influence of *in utero* drug exposure on cSLE and other childhood autoimmune diseases.

The main strength of our study was the child-parent connection provided by the nationwide population-based cohort. The accuracy of the integrated databases minimized the selection and referral biases. The large sample size in our databases offered sufficient power to evaluate rare events of cSLE. All diagnoses of SLE and other systemic autoimmune diseases for the participants were confirmed by the relevant CICs, which ensured the accuracy of the diagnoses. The possibilities of loss to follow-up and movement out of the system are rare because of the mandatory National Health Insurance program, especially for pregnant women and children. Furthermore, we evaluated the influence of concomitant paternal immune diseases on the risk of cSLE, thus indicating the novelty of our study.

This study has several limitations. Firstly, the follow-up period of our study was 13 years, and a substantial number of adolescents with SLE could not be included; therefore, we may have underestimated the incidence of pediatric SLE. Secondly, we were unable to access the records regarding serum levels of immune markers (e.g., complement or autoantibodies) and urine protein levels. Therefore, we could not analyze the impact of maternal lupus activity on outcomes. Thirdly, potential unmeasured confounders, such as smoking, physical stress during pregnancy, and sunlight exposure, were not available in the NHIRD. Therefore, we could not investigate the potential effects of epigenetic factors on cSLE; however, we adjusted urbanization and income levels to mitigate this bias because it is possible that socioeconomic status was considered a surrogate for environmental factors. Fourthly, potential misclassification could have existed in an observational study; however, we considered cases to be true SLE cases only when participants had both SLE diagnosis codes and relevant CICs, and we limited maternal SLE to pregnant women with diagnosis codes within 4 years before the index date to mitigate the possibility of misclassification. Fifthly, 567,299 births were excluded from analyses; these births comprise approximately 21.1% of the entire birth population during the study period. Among identifiable mother–child relationships, there were 872 (0.15%) singletons of women with SLE and 566,418 (99.85%) singletons of women without SLE. The distribution of children of women with and without SLE was similar among the eligible population and those excluded births; therefore, the impact on outcome estimates was minimal. Finally, this nationwide study was conducted in Taiwan, and the results have potentially limited generalizability to other populations.

## Conclusion

Rheumatologists commonly perform fertility counseling for married couples with SLE regarding whether their children could be at risk for adverse perinatal outcomes or childhood health. A better understanding of the risk of childhood-onset immune diseases would help rheumatologists provide useful familial counseling for anxious couples with SLE. This large-scale cohort study demonstrated that children of women with SLE are at increased risk for early-onset cSLE. Our study results highlight the significance of genetic predisposition to cSLE and the susceptibility to both maternal and paternal diseases. During routine care for individuals with SLE and SS, rheumatologists should remind them of the potential risks that could affect their children.

## Data availability statement

The original contributions presented in the study are included in the article/**Supplementary Material**. Further inquiries can be directed to the corresponding authors.

## Ethics statement

The studies involving human participants were reviewed and approved by The Institutional Review Board of the National Cheng Kung University Hospital. Written informed consent from the participants’ legal guardian/next of kin was not required to participate in this study in accordance with the national legislation and the institutional requirements.

## Author contributions

Conceptualization: C-HW, C-AC, and C-TW. Methodology: C-HW and S-HL. Software: S-HL. Validation: C-TW and P-LK. Formal analysis: C-HW and C-AC. Investigation: S-HL. Resources: P-LK and C-CS. Data curation: C-AC and C-TW. Writing—original draft preparation: C-HW. Writing—review, and editing: C-AC and C-CS. Visualization: C-HW and S-HL. Supervision: P-LK and C-CS. Project administration: P-LK and C-CS. Funding acquisition: C-HW and C-AC. All authors have read and agreed to the published version of the manuscript.

## Funding

This work was supported by the National Cheng Kung University Hospital (NCKUH-11004037 and NCKUH-11006005) and the Ministry of Science and Technology (MOST 110-2314-B-006-092-MY3). The funders had no role in the design and conduct of the study; collection, management, analysis, and interpretation of the data; preparation, review, or approval of the manuscript; and decision to submit the manuscript for publication.

## Acknowledgments

We are grateful to Mrs. Wan-Ni Chen from the Biostatistics Consulting Center, Clinical Medicine Research Center, National Cheng Kung University Hospital, for providing statistical consulting services.

## Conflict of interest

The authors declare that the research was conducted in the absence of any commercial or financial relationships that could be construed as a potential conflict of interest.

## Publisher’s note

All claims expressed in this article are solely those of the authors and do not necessarily represent those of their affiliated organizations, or those of the publisher, the editors and the reviewers. Any product that may be evaluated in this article, or claim that may be made by its manufacturer, is not guaranteed or endorsed by the publisher.
