# Supplementary material for: Increased risk of early-onset childhood systemic lupus erythematosus for children born to affected parents: A nationwide child-parent cohort study
Source: Front Immunol. 2022 Sep 5;13:966809. doi: 10.3389/fimmu.2022.966809 (PMC9483159; doi:10.3389/fimmu.2022.966809)
Supplement: Supplementary file 1 [file DataSheet_1.docx]

Supplementary Material

**Supplementary Table S1. *International Classification of Diseases, Ninth Revision, Clinical Modification* (ICD-9-CM) and *International Classification of Diseases, Tenth Revision, Clinical Modification* (ICD-10-CM) codes of procedures and diseases**

| **Variables** | **ICD-9-CM codes** | **ICD-10-CM codes** |
| --- | --- | --- |
| **Diseases** |  |  |
| Systemic lupus erythematosus | 710.0 | M32.1–M32.9 |
| Sjögren’s syndrome | 710.2 | M350x |
| Rheumatoid arthritis | 714.0  714.1  714.2  714.8x1 | M05x, M060x, M068x, M069 |
| PIH/preeclampsia/eclampsia | 642.3–7 | O13, O16, O14, O15 |
| Gestational diabetes | 648.0, 648.8 | O24.4xx, O99.810 |
| Graves’ disease | 242.0 | E0500, E0501 |
| Hashimoto thyroiditis | 245.2 | E063 |
| Type 1 diabetes mellitus | 250.x1, 250.x3 | E10x |
| **Procedures** |  |  |
| Normal spontaneous delivery | 650 | O80 |
| Cesarean delivery | 669.7 | O82 |

PIH: Pregnancy-induced hypertension.

**Supplementary Table S2.** Crude and adjusted hazard ratios of risk factors for early-onset cSLE

|  | **Adjusted HR^a^**  **(95% CI)** | ***p* value** |  | **Fully adjusted HR^b^**  **(95% CI)** | ***p* value** |  |  |  |  |
| --- | --- | --- | --- | --- | --- | --- | --- | --- | --- |
|  |  |  |  |  |  |  |  |  |  |
| **Maternal SLE** | 4.81 (2.17–10.65) | <0.001 |  | 4.65 (2.11–10.24) | <0.001 |  |  |  |  |
| **Offspring sex** | | |  |  | |  | |  | |
| Male | reference |  |  | reference |  |  |  |  |  |
| Female | 2.46 (2.03–2.98) | <0.001 |  | 2.46 (2.03–2.97) | <0.001 |  |  |  |  |
| **Gestational age category** | | |  |  | |  | |  | |
| <31 | 1.20 (0.39–3.76) | 0.750 |  | 1.11 (0.35–3.51) | 0.862 |  |  |  |  |
| 31–33 | 0.38 (0.35–2.66) | 0.292 |  | 0.35 (0.35–2.47) | 0.292 |  |  |  |  |
| 34–36 | 1.25 (0.88–1.77) | 0.213 |  | 1.21 (0.85–1.75) | 0.299 |  |  |  |  |
| ≥37 | reference |  |  | reference |  |  |  |  |  |
| **Birthweight for gestational age** | | |  |  |  |  |  | |  |
| SGA | 1.08 (0.80–1.46) | 0.596 |  | 1.06 (0.79–1.43) | 0.696 |  |  |  |  |
| AGA | reference |  |  | reference |  |  |  |  |  |
| LGA | 1.11 (0.84–1.47) | 0.456 |  | 1.11 (0.84–1.47) | 0.475 |  |  |  |  |
| **Obstetric complications** |  |  |  |  |  |  |  |  |  |
| PIH/preeclampsia/eclampsia | N/A |  |  | 1.52 (1.01–2.30) | 0.047 |  |  |  |  |
| GDM | N/A |  |  | 0.97 (0.74–1.27) | 0.823 |  |  |  |  |
| **Concomitant autoimmune diseases** | | |  |  | |  | |  | |
| Paternal SLE | 12.95 (2.66–63.16) | 0.002 |  | 12.94 (2.66–62.98) | 0.002 |  |  |  |  |
| Maternal Sjögren’s syndrome | 3.12 (1.39–7.00) | 0.006 |  | 3.14 (1.40–7.02) | 0.005 |  |  |  |  |
| Paternal Sjögren’s syndrome | 4.68 (1.57–13.93) | 0.006 |  | 4.71 (1.59–13.99) | 0.005 |  |  |  |  |
| Maternal Rheumatoid arthritis | 1.06 (0.25–4.43) | 0.942 |  | 1.06 (0.25–4.43) | 0.936 |  |  |  |  |
| Maternal Graves’ disease | 1.33 (0.55–3.20) | 0.531 |  | 1.31 (0.54–3.16) | 0.548 |  |  |  |  |
| **Maternal age group (years)** |  |  |  |  |  |  |  |  |  |
| <20 | 0.81 (0.34-1.97) | 0.649 |  | 0.82 (0.34-1.98) | 0.657 |  |  |  |  |
| 20-24 | 1.14 (0.83-1.55) | 0.419 |  | 1.14 (0.84-1.56) | 0.408 |  |  |  |  |
| 25-29 | reference |  |  | reference |  |  |  |  |  |
| 30-34 | 1.36 (1.07-1.73) | 0.011 |  | 1.36 (1.07-1.73) | 0.012 |  |  |  |  |
| 35-39 | 1.72 (1.23-2.39) | 0.001 |  | 1.70 (1.23-2.36) | 0.001 |  |  |  |  |
| ≥40 | 0.76 (0.26-2.08) | 0.586 |  | 0.73 (0.26-2.02) | 0.545 |  |  |  |  |
| **Paternal age group (years)** |  |  |  |  |  |  |  |  |  |
| <24 | 0.91 (0.56-1.49) | 0.704 |  | 0.91 (0.56-1.49) | 0.705 |  |  |  |  |
| 25-29 | reference |  |  | reference |  |  |  |  |  |
| 30-34 | 0.87 (0.67-1.12) | 0.277 |  | 0.87 (0.67-1.12) | 0.277 |  |  |  |  |
| 35-39 | 0.93 (0.69-1.26) | 0.643 |  | 0.93 (0.69-1.26) | 0.637 |  |  |  |  |
| 40-44 | 0.77 (0.49-1.19) | 0.238 |  | 0.76 (0.49-1.19) | 0.231 |  |  |  |  |
| 45-49 | 0.69 (0.30-1.57) | 0.373 |  | 0.68 (0.30-1.56) | 0.364 |  |  |  |  |
| ≥50 | 0.74 (0.18-3.06) | 0.679 |  | 0.74 (0.18-3.05) | 0.673 |  |  |  |  |
| **Family income level** |  |  |  |  |  |  |  |  |  |
| Dependent or <Q1 | reference |  |  | reference |  |  |  |  |  |
| Q1 to <Q2 | 0.89 (0.67-1.19) | 0.429 |  | 0.89 (0.67-1.19) | 0.431 |  |  |  |  |
| Q2 to <Q3 | 1.02 (0.77-1.36) | 0.887 |  | 1.02 (0.77-1.36) | 0.880 |  |  |  |  |
| ≥Q3 | 1.23 (0.93-1.64) | 0.151 |  | 1.24 (0.93-1.65) | 0.140 |  |  |  |  |
| **Urbanization level** |  |  |  |  |  |  |  |  |  |
| Level 1 or 2 | reference |  |  | reference |  |  |  |  |  |
| Level 3 or 4 | 1.06 (0.74-1.54) | 0.740 |  | 1.07 (0.74-1.54) | 0.732 |  |  |  |  |
| Level 5 | 0.81 (0.56-1.16) | 0.249 |  | 0.81 (0.56-1.17) | 0.257 |  |  |  |  |
| Level 6 | 0.70 (0.49-1.00) | 0.052 |  | 0.70 (0.49-1.01) | 0.055 |  |  |  |  |
| Level 7 | 1.04 (0.73-1.49) | 0.839 |  | 1.04 (0.73-1.49) | 0.823 |  |  |  |  |

SLE, systemic lupus erythematosus; SGA, small for gestational age; AGA, appropriate for gestational age; LGA, large for gestational age; PIH, pregnancy-induced hypertension; GDM, gestational diabetes mellitus; CI, confidence interval; HR, hazard ratio; N/A, not applicable.

^a^Adjusted HRs were estimated using partially adjusted Cox proportional hazard regression models but excluding the variable of obstetric complications (PIH/preeclampsia/eclampsia, and gestational diabetes); each covariate was adjusted for variables listed in the table, except for the variable itself.

^b^Adjusted HRs were estimated using fully adjusted Cox proportional hazard regression models; each covariate was adjusted for variables listed in the table, except for the variable itself.

**Supplementary Table S3.** Sensitivity analysis by defining the index as 2 years after childbirth

|  | **Children of mothers without SLE** | **Children of mothers with SLE** | ***p* value** |
| --- | --- | --- | --- |
| **Median follow-up duration, (25%-75% IQR), years** | 7.13 (4.41–9.58) | 7.02 (4.25–9.62) | 0.142 |
| **Early-onset cSLE** | 431 (0.02) | 8 (0.20) | <0.001 |
| **Disease onset age, mean (S.D.), years** | 8.07 (2.46) | 8.61 (3.04) | 0.587 |
| **Incidence rate of early-onset cSLE (1000 person-years)** | 0.034 | 0.294 |  |
| **Incidence rate ratio of early-onset cSLE (95% CI)** | 1.0 (reference) | 8.67 (4.31–17.45) | <0.001 |
| **Adjusted HR for cSLE** | 1.0 (reference) | 5.43 (2.40–12.26) | <0.001 |

IQR, interquartile range; SLE, systemic lupus erythematosus; cSLE, childhood-onset SLE; SD, standard deviation; CI, confidence interval.
